# Secondary diabetes mellitus in acromegaly

**DOI:** 10.1007/s12020-023-03339-1

**Published:** 2023-03-08

**Authors:** Melpomeni Moustaki, Stavroula A. Paschou, Paraskevi Xekouki, Kalliopi Kotsa, Melpomeni Peppa, Theodora Psaltopoulou, Sophia Kalantaridou, Andromachi Vryonidou

**Affiliations:** 1 Department of Endocrinology and Diabetes Center, Hellenic Red Cross Hospital, Athens, Greece; 2 Endocrine Unit and Diabetes Center, Department of Clinical Therapeutics, Alexandra Hospital, School of Medicine, National and Kapodistrian University of Athens, Athens, Greece; 3 Department of Endocrinology and Diabetes, University General Hospital of Heraklion, School of Medicine, University of Crete, Heraklion, Greece; 4 Endocrine Unit and Diabetes Center, First Department of Internal Medicine, AHEPA University Hospital, School of Medicine, Aristotle University of Thessaloniki, Thessaloniki, Greece; 5 Endocrine Unit and Diabetes Center, Second Department of Internal Medicine, Attikon University Hospital, School of Medicine, National and Kapodistrian University of Athens, Athens, Greece; 6 3rd Department of Obstetrics and Gynecology, Attikon University Hospital, School of Medicine, National and Kapodistrian University of Athens, Athens, Greece

**Keywords:** Acromegaly, Growth hormone, Secondary diabetes mellitus, Insulin resistance, IGF-1 resistance, Pasireotide-induced hyperglycemia

## Abstract

Secondary diabetes mellitus (DM) is a common complication of acromegaly, encountered in up to 55% of cases. Vice versa, the prevalence of acromegaly is markedly higher in cohorts of patients with type 2 DM (T2DM). The presence of secondary DM depends primarily on acromegaly status and is associated with increased cardiovascular morbidity, malignancy rate and overall mortality. The principal pathophysiologic mechanism is increased insulin resistance due to excessive lipolysis and altered fat distribution, reflected at the presence of intermuscular fat and attenuated, dysfunctional adipose tissue. Insulin resistance is ascribed to the direct, diabetogenic effects of growth hormone (GH), which prevail over the insulin-sensitizing effects of insulin-like growth factor 1 (IGF-1), probably due to higher glucometabolic potency of GH, IGF-1 resistance, or both. Inversely, GH and IGF-1 act synergistically in increasing insulin secretion. Hyperinsulinemia in portal vein leads to enhanced responsiveness of liver GH receptors and IGF-1 production, pointing towards a mutually amplifying loop between GH-IGF-1 axis and insulin. Secondary DM occurs upon beta cell exhaustion, principally due to gluco-lipo-toxicity. Somatostatin analogues inhibit insulin secretion; especially pasireotide (PASI) impairs glycaemic profile in up to 75% of cases, establishing a separate pathophysiologic entity, PASI-induced DM. In contrast, pegvisomant and dopamine agonizts improve insulin sensitivity. In turn, metformin, pioglitazone and sodium-glucose transporters 2 inhibitors might be disease-modifying by counteracting hyperinsulinemia or acting pleiotropically. Large, prospective cohort studies are needed to validate the above notions and define optimal DM management in acromegaly.

## Introduction

Acromegaly is a rare disease, with a reported prevalence of 0.006%, being characterized by growth hormone (GH) and insulin-like-growth factor (IGF-1) excess, caused, in ~99% of cases, by a GH-secreting pituitary adenoma [1]. The clinical sequalae of acromegaly stem from hormonal effects in target tissues and mass effects of the pituitary adenoma. The former group includes acral, facial and soft tissue overgrowth, arthritis, carpal tunnel syndrome, hyperhidrosis, visceromegaly, hyperglycemia, hypertension, cardiomyopathy, obstructive sleep apnea and tumorigenesis [1, 2].

The increased prevalence of hyperglycemia in acromegaly has been recognized from early case series in 1950s and 1960s [3, 4]. Actually, the rates of prediabetes and diabetes mellitus (DM) range between 5–33% [5–8] and 14.6–54.5% respectively [5–17] in cohort and national registry studies, while the first international acromegaly database including 3173 patients (Liege Acromegaly Survey Database) indicates 27.5% prevalence of DM at acromegaly diagnosis [18]. The fact that, in the majority of cases, hyperglycemia precedes acromegaly diagnosis [5, 19] reflects the characteristic time lag between clinical presentation and diagnosis of the latter [1].

Vice versa, the prevalence of acromegaly is at least 100-fold higher in cohorts of diabetic or prediabetic patients compared to general population, ~0.6–0.13% [20, 21]. Indeed, employing systematic biochemical screening followed by pituitary magnetic resonance imaging (MRI), when indicated, in hospitalized patients with type 2 diabetes mellitus (T2DM) and unsuspected acromegaly, resulted in picking up 2 cases of acromegaly. Their clinical profile was mild in clinical and biochemical terms, both beared pituitary microadenomas and, interstingly, excibited severe macroangiopathy in the absence of microangiopathy. These data imply that screening T2DM patients for acromegaly, especially those with predominant macroangiopathy, might be a useful strategy of depicting the disease at earlier stages, in which remission post-treatment is more likely [21].

Secondary DM in acromegaly is identified as a specific type of DM [22]; expectedly, its presence depends on acromegaly activity [8, 23, 24] and duration [6]. In contrast, acromegaly treatment and biochemical control decrease the hazard of new-onset DM [23, 24] and might lead to remission of pre-existing DM [7, 10]. In accordance with these observations, patients with prediabetes and DM are demonstrated to have higher IGF-1 [5–7, 13, 17, 25, 26] and GH levels than those with normal glucose tolerance (NGT); in addition, glycemic status might be predicted by IGF-1 level post-operatively [7]. Other risk factors for hyperglycemia in acromegaly include age [6, 11] body mass index (BMI) [5, 6, 11], hypertension [8, 11, 17], female sex [8, 17], and family history of DM [5, 8].

The presence of secondary DM aggravates the clinical presentation and prognosis of acromegaly. Specifically, it is shown to increase overall mortality [27], malignancy rate [25], as well as cardiovascular morbidity and mortality [27], compared to acromegaly alone. The latter derives principally from worsening of hypertrophic cardiomyopathy [28]; this is reflected at recent, 3-dimensional, speckle-tracking, echocardiographic data showing exacerbation of left ventricular deformation [29]. Last but not least, secondary DM is associated with increased prevalence of vertebral fractures [30], postoperative weight and fat gain [31] and worse quality of life [32].

Despite that secondary DM in acromegaly is common and clinically relevant, the underlying pathophysiology is not entirely understood. Furthermore, the medications used in treatment of acromegaly may affect insulin sensitivity or secretion per se. Finally, DM management in acromegaly is scarcely studied.

In this article, we aim to present the relationship between GH, IGF-1, insulin signaling and glucose homeostasis and how acromegaly affects it. Moreover, to discuss how medical treatment for acromegaly and DM join this complex pathophysiological process according to the available experimental and clinical evidence.

## Insulin sensitivity

Acromegaly is a state of insulin resistance. According to a recent, large metanalysis (492 patients with acromegaly vs. 12,745 population group), homeostasis model assessment for insulin resistance (HOMA-IR) in treatment-naïve patients is higher than in the reference population [33]. Consistently, cohort studies demonstrate decreased insulin sensitivity (Si) during intravenous glucose tolerance test [34], decreased glucose infusion rate during hyperinsulinemic euglycemic clamp [35], and decreased homeostasis model assessment for insulin sensitivity [5]. These insulin sensitivity indices are impaired not only in patients with secondary DM but also in patients with NGT [5, 33, 34, 36], suggesting that insulin resistance is the primary pathophysiologic defect of glucose metabolism in acromegaly.

Insulin resistance is amenable upon successful acromegaly treatment, shown by significant improvements in the above markers post-surgery [33, 35, 36]. Interestingly, HOMA-IR decreases as early as 9 days postoperatively [36], reflecting exclusively the previous effect of acromegaly in insulin sensitivity. The etiologic association between insulin resistance and acromegaly is mirrored by the correlations between insulin sensitivity markers and IGF-1 level [5, 26, 35, 36]; however, similar correlations with GH level are either not demonstrated [5, 35, 36] or weaker [26].

Insulin resistance presents gender dimorphism with female predominance, concerning especially post-menopausal women and being possibly related to higher visceral adipose tissue (VAT) in comparison to men. This is opposite to general population, where men have higher VAT [37]. Finally, BMI is an independent predictor of insulin resistance, as in “wild-type” T2DM [5].

### Growth hormone (GH)

GH is a counter-regulatory hormone that antagonizes the effects of insulin. Lipolysis is the principal operating mechanism [38], as evidenced by restoration of total body insulin sensitivity in GH-exposed subjects upon pharmacologic blockade of lipolysis with acipimox [39]. The relationship between increased free fatty acids (FFAs) and insulin resistance at cellular level has been recognized from early studies, and our pathophysiological perspective has evolved from Randle hypothesis [40] to the theory of post-receptor inhibition of insulin signaling by FFAs [41].

The different sensitivity of subcutaneous adipose tissue (SAT), VAT and intermuscular adipose tissue (IMAT) to GH’s lipolytic effect, leads to a unique form of lipodystrophy, characterized by lower total adipose tissue (TAT), SAT, and VAT yet higher IMAT [42, 43]. In contrast, “wild-type” T2DM and obesity are associated with decreased GH secretion, possibly due to hyperglycemia-increased hypothalamic somatostatin tone, which, in turn, might increase VAT [44].

#### Muscle

Early studies in 1960s and 1970s have shown that GH administration inhibits glucose uptake in parallel with increasing FFA uptake in muscle [45, 46]. Human and rodent data from magnetic resonance spectroscopy during hyperinsulinemic euglycemic clamp post lipid/heparin infusion show that increased FFA delivery in the myocyte activates protein kinase C theta, which, in turn, favors serine over tyrosine phosphorylation of insulin receptor substrate 1 (IRS-1), resulting into diminishing phosphatidylinositol-3-kinase (PI3K) activity and subsequent insulin-stimulated glucose-transport [41, 47, 48]. Nevertheless, no effect in IRS-1-PI3K-protein kinase B-glucose transporter-4 (GLUT-4) signaling pathway is demonstrated in another study post short-term GH administration [49]. The latter study though, included healthy human subjects, whose FFA level suppressed during hyperinsulinemic euglycemic clamp contrary to patients with acromegaly, whose FFA level remains unsuppressed despite compensatory hyperinsulinemia [50]. Furthermore, GH increases FFA oxidation and shifts glucose metabolism to non-oxidative [50, 51].

Clinical data from patients with acromegaly indicate a negative correlation between intramyocellular lipid and insulin sensitivity [52]. Similar correlations between IMAT and insulin resistance are proven in “wild-type” obesity and T2DM, non-diabetic Afro-American women, and obese human immunodeficiency virus infected women [53–55].

#### Adipose tissue

GH inhibits lipogenesis and induces lipolysis in adipose tissue. The former is mediated via inhibition of lipoprotein lipase [56] and fatty acid synthase expression [57], while the latter involves enhanced hormone-sensitive lipase expression, adenylate cyclase function and responsiveness to β-adrenergic signaling [58, 59]. In terms of glucose metabolism, GH is demonstrated to increase the expression of p85 subunit of PI3K in mice, a negative regulator of the latter [60], leading directly to decreased glucose uptake.

Furthermore, GH excess perturbs adipokine expression. Leptin is decreased due to SAT depletion; expectedly, it increases post-treatment alongside SAT [61, 62]. Adiponectin is shown to be decreased in mice with GH excess [60] and to increase post-operatively in acromegalic patients [63]. This decrease in adiponectin, otherwise paradoxical considering decreased VAT [64], is probably caused by transcriptional downregulation upon GH binding to signal transducer and activator of transcription 5 site of adiponectin gene promoter [65]. On the contrary, nicotinamide phosphorybosultransferase, another adipokine also known as visfatin, is increased, favoring inflammation and FFA oxidation over glycolysis [66, 67]. Finally, GH upregulates the expression of a variety of inflammatory cytokines, including monocyte chemotactic protein 1, vascular endothelial growth factor-A and interleukin-6 in VAT and SAT [68].

#### Liver

Data from transgenic mice overexpressing GH demonstrate markedly increased basal levels of IRS-1 phosphorylation and PI3K activity, which fail to further increase post exogenous insulin administration in their portal vein. These findings indicate that both hyperinsulinemia and direct effects of GH to the insulin receptor (IR), lead to maximal basal phosphorylation of IRS-1 and PI3K, inducing insensitivity to further insulin boluses. In addition, these mice have decreased basal IR level, owing to downregulation by hyperinsulinemia; this consists a further mechanism of insulin resistance in this setting [69]. Additionally, GH stimulates glucagon secretion from alpha pancreatic cells in vitro, which may further stimulate liver glucogeogenesis in this setting [70, 71]; however, this has not been demonstrated in a clinical study with acromegalic patients [72].

### Insulin-like growth factor 1 (IGF-1)

As denoted by its name, IGF-1 exerts insulin-sensitizing effects [73]. The administration of recombinant IGF-1 in patients with “wild-type” T2DM leads to improvement of glycemic control and insulin sensitivity [74]. Phylogeny studies establish that proinsulin and IGF-1 have evolved from a common ancestor gene; the function of insulin and IGF -1 diverge in vertebrates between metabolic and mitogenic respectively [75]. Likewise, IGF-1 receptor (IGF-1R) is closely related to the IR; they are both members of the subclass of transmembrane tyrosine kinase receptors [76]. IGF-1R is expressed in muscle [77] and adipose tissue [78] but not in liver [79].

#### Muscle

Muscle expresses both IGF-1Rs and hybrid receptors, i.e., heterodimers consisting of one subunit of IR and one subunit of IGF-1R. Upon IGF-1 stimulation, hybrid receptors activate GLUT-4 translocation and facilitate glucose uptake in healthy subjects but not in “wild-type” obese or diabetic subjects [77]. Of note, these subjects have increased abundance of hybrid receptors [80, 81]. These findings suggest that insulin resistance goes hand-in hand with IGF-1 resistance in muscle.

#### Adipose tissue

IGF-1R is expressed in adipose tissue, however, as shown in vitro, IGF-1 stimulates glucose-transport predominantly through the IR [78]. As in muscle, hybrid IR/IGF-1R receptors are formed, and their abundance is increased in “wild-type” T2DM; of note, it is proposed that hybrid receptors could contribute to insulin resistance by binding IGF-1 with higher affinity than insulin [82]. Contrary to GH, IGF-1 upregulates the expression of adiponectin [83].

#### GH vs. IGF-1: What determines the net effect in insulin sensitivity?

It is evident that the diabetogenic effect of GH prevails over the insulin-sensitizing effect of IGF-1. Acromegalic patients with impaired glucose tolerance (IGT) and DM have lower IGF-1/GH ratio than those with NGT in one cohort study, offering a mathematically plausible explanation to this phenomenon [84]. However, considering that insulin resistance indices are correlated with IGF-1 and not with GH in the majority of studies [5, 26, 35, 36], the etiology of predominance of GH effect appears to be more complicated.

Looking at transgenic animal models, liver IGF-1 deficient mice (LID) have subsequent 4-fold increase in GH secretion and are severely insulin-resistant [85]. When crossed with GH antagonist mice (GHa), LID + GHa mice demonstrate enhanced insulin sensitivity in muscle and adipose tissue, despite being more profoundly IGF-1 deficient. Therefore, the diabetogenic potential of GH appears to supersede the insulin-sensitizing capacity of IGF-1 [86].

Furthermore, acromegaly may induce IGF-1 resistance due to IGF-1R downregulation or desensitization in the setting of chronic exposure to IGF-1 and concomitant hyperinsulinemia. Meanwhile, Clotho, a potent inhibitor of both IR and IGF-1R via interruption of their tyrosine phosphorylation, has been recently found to be increased in acromegaly, thus, it may also contribute to IGF-1 resistance. IGF-1 resistance is selective for the metabolic function of IGF-1 receptor [87].

## Insulin secretion

Acromegaly is accompanied by fasting and post-prandial hyperinsulinemia [38, 88]. From early studies, it has become evident that the latter is not only a consequence insulin resistance, as the degree of hyperglycemia does not fully account for the the augmented secretion of insulin [88]. Indeed, both GH and IGF-1 have been shown to have direct insulinotropic effects in beta cells [70, 71, 89]. GH administration acutely stimulates insulin release both in vivo and in vitro [70]. This effect is essential for glucose-stimulated first-phase insulin secretion [90], but leads to exaggerated insulin responses both to hyperglycemic and non-hyperglycemic stimuli under circumstances of GH excess, which may last up to 5 h postprandially [88]. Acording to data ex vivo, IGF-1 also enhances glucose- and arginine-stimulated insulin secretion, via promoting exocytosis of beta cell [89]. Furthermore, GH is shown to induce insulin gene expression and biosynthesis, as well as beta cell proliferation in vitro [91] and ex vivo [90]. The underlying mechanisms principally involve activation of Janus kinase/signal transducers and activators of transcription (JAK/STAT) pathways and insulin receptor substrate 2 (IRS-2) activation [90, 91]. Similarly, IGF-1 has been speculated to have similar proliferative proterties [91], yet no effect in insulin content and beta cell mass was demonstrated in the above study ex vivo [89].

Hyperinsulinemia manages to initially compensate for the increased insulin resistance, maintaining NGT. IGT and DM occur when this compensation can no longer be achieved. Indeed, patients with acromegaly and NGT demonstrate higher homeostasis model assessment of beta cell function (HOMA-β) and insulin response to glucose and arginine than those with prediabetes/DM [5, 34, 72, 84]. Apart from defining glycemic status preoperatively, beta cell function status (assessed by c-peptide, HOMA-β, disposition index) is the key predictor of alteration in glucose metabolism post-operatively [92, 93].

Impaired insulin secretion derives from beta cell exhaustion and gluco-lipo-toxicity, similarly to “wild-type” T2DM. Furthermore, glucose-dependent insulinotropic peptide (GIP) level is shown to be increased yet inversely related with HOMA-β, pointing towards GIP resistance as an additional mechanism [94]. Severe insulin deficiency alongside with excessive lipolysis can lead to diabetic ketoacidosis (DKA) in acromegaly, with several cases reported in the literature [95–98].

## GH-IGF-1/insulin interplay

The relationship between IGF-1 and insulin is mutually beneficial: IGF-1 increases insulin sensitivity and secretion, and, in turn, insulin enhances IGF-1 secretion via increasing the responsiveness of liver GH receptors (GHR). States of insulin deficiency, like type 1 DM (T1DM) or decompensated T2DM are accompanied by impaired IGF-1 synthesis [99, 100]. IGF-1 level increases upon initiation of insulin therapy in T1DM [99] and glycated hemoglobulin (HbA1c) improvement in T2DM [100]. In both DM types, IGF-1 secretion is best correlated c-peptide level [99, 100], highlighting the importance of endogenous insulin for IGF-1 production, probably due to being related with higher intraportal insulin concentrations. The latter is proven in a study of pancreas-transplanted T1DM patients; specifically, patients with portal-vein drainage grafts exhibit higher IGF-1 and lower GH levels than those with systemic-vein drainage grafts [101].

Poor glycemic control has challenged acromegaly diagnosis due to low/normal IGF-1 level in 2 case studies; similarly to “wild-type” T2DM, IGF-1 also increases post insulin-treatment [102, 103] in secondary DM. On the contrary, analysis from ACROSTUDY reveals that patients with DM require higher pegvisomant (PEGV) doses to normalize IGF-1 [104]; this is explained by the fact that mean HbA1c among diabetic patients in this study is 7%, reflecting an hyperinsulinemic DM phenotype, due to either endogenous or exogenous insulin [104].

## The impact of medical treatment of acromegaly in glucose metabolism

Unlike surgery or radiotherapy, which affect glucose metabolism only indirectly, via targeting hormonal excess or inducing hypopituitarism, the medical treatment of acromegaly affects insulin sensitivity or secretion in direct manner.

### Somatostatin receptor ligands (SRLS)

Somatostatin is produced in hypothalamus, pancreatic islets and the upper gastrointestinal tract. Apart from suppressing GH secretion by activating somatostatin receptor subtype 2 and 5 (SSR2, SSR5) in somatotroph adenomas, it suppresses glucagon and insulin secretion by activating somatostatin receptor subtype 1(SSR1), SSR2 and SSR5 in alpha and beta cells. Although all the above receptors are axpressed in both alpha and beta cells, the former are more abundant in SSR2 and the latter in SSR1 and SSR5 [105].

#### First-generation somatostatin receptor ligands (SRLS): ocreotide and lanreotide

These somatostatin receptor ligands (SRLS) have specificity for SSR2 [106]. According to a metanalysis, first-generation SRLS lead to minor worsening of glucose homeostasis, including decreased glucose tolerance and fasting insulin level, with no change in fasting plasma glucose (FPG) or HbA1c [107]. In addition, a comparative study between equally well-controlled patients on either lanreotide or post-pituitary surgery reveals no difference in HbA1c [108]. Consistently, 3 single-center studies indicate similar percentages of deterioration and improvement of glucose tolerance on lanreotide monotherapy [109–111]. As reflected in one of them, the overall impact of SRLS treatment on glucose metabolism for each patient depends on the dynamic interaction between improvement in insulin sensitivity and deterioration of insulin secretion, reflected at oral glucose tolerance test (OGTT) insulin sensitivity index and HOMA-β respectively [109]. The former is principally driven by GH reduction per se; consistently, the key predictor of glycemic deterioration on SRLS treatment in all these studies is suboptimal acromegaly control [109–111]. The latter is caused by SSR2-mediated inhibition of insulin secretion; on the other hand, the SSR2-mediated glucagon suppression might meanwhile improve insulin sensitivity. [111]. Furthermore, patients with pre-existing IGT or DM are more prone to glycemic deterioration during treatment with SRLS [110]. Non-diabetic (at baseline) and biochemically controlled on SRLS patients show an overall 44% deterioration in glycemic status at 6-year follow up in another study, which is fully reversible upon SRLS discontinuation and surgical cure of acromegaly [112]. This study, despite small, is important because it indicates that in the absence of confounding factors such as uncontrolled acromegaly or pre-existing DM, the net direct effect of SRLS in glucose metabolism is adverse, implying that the inhibition of insulin secretion supercedes this of glucagon. Finally, while opposing effects in insulin sensitivity and secretion self-adjust in most cases, enhanced insulin sensitivity in insulin-treated DM patients can increase the risk of hypoglycemia as recently shown in a case report [113].

#### Second generation somatostatin receptor ligand: Pasireotide (PASI)

Pasireotide (PASI) binds multiple subtypes of somatostatin receptors, bearing high affinity for SSR5 [106] and is associated with markedly higher rates of hyperglycemia compared to first-generation SRLS [114, 115]. According to the analysis of clinical trials data, the rates of hyperglycemia and DM on PASI treatment ~41.5–42.4% and 23.6–24.2% respectively [116, 117], while 65.6–75.3% of patients develop or experience worsening of existing hyperglycemia [118]. Notably, the majority of patients in the above trials are diabetic or prediabetic at baseline [116–118] and the main effect of PASI is to convert prediabetes to DM [116, 117]. Glycemic deterioration presents early, approximately at 3 months, but remains stable thereafter [117, 119]. Based on real-world data, the mean increases in FBG and HbA1c are 13.06 mg/dl and 0.42% respectively, with 23.1 % of patients having deranged glycemic control [119]. Similarly to first-generation SRLS, PASI-related hyperglycemia is reversible upon drug discontinuation [120].

According to data from healthy volunteers, PASI inhibits insulin secretion not only by its direct effect in beta cells, but also indirectly, by decreasing incretin levels, GIP and glucagon-like peptide 1 (GLP-1), possibly by activating SSR5 on K and L cells [121]. Interestingly though, AP102, a new SRL with high affinity for both SSTR2 and SSTR5 is shown to have a neutral effect in glucose metabolism in rats. The two agents have been compared head to head in an in vivo study in rats over a 28-day treatment period and they have been both demonstrated to inhibit insulin secretion. Their main difference is that AP102 does not inhibit GLP-1 secertion. Moreover, AP102-induced glucagon suppression remains stable for the whole treatment period. In contrast, PASI-induced glucagon suppression is reversed post 21 days of treatment, increasing glucagon/insulin ratio over the last 7 days, further aggravating glucose tolerance [122]. These observations suggest that diabetogenic effects of PASI might involve other somatostatin receptors activated by PASI but not by AP102, such as SSR1 and somatostatin receptor subtype 3 (SSR3); of note, both are abundantly expressed in beta cells [105, 122]. Finally, glucose outcomes on treatment with PASI are correlated with age, HbA1c/glycemic status at baseline, and history of hypertension or dyslipidemia [118, 119].

### Pegvisomant (PEGV)

Pegvisomant (PEGV) is a pegylated human GHR antagonist. Unlike SRLS, it is associated with favorable glucose homeostasis outcomes. In particular, short-term PEGV administration decreases endogenous glucose production in liver, alongside with suppression of lipolysis [123]. Consistently, analyses of Spanish and international data of ACROSTUDY show that PEGV decreases FPG in patients with secondary DM [124, 125] as well as the percentage of patients with IGT (6.4% vs. 11.2%) [125].

As previously analyzed, GH is the protagonist in mediating insulin resistance; therefore, the blockade of its action would improve insulin sensitivity regardless of concomitant IGF-1 decrease. Laron syndrome is an autosomal recessive disorder characterized by inactivating GHR mutation, therefore it could serve as a natural analogue of PEGV. Indeed, Equadorian adults with Laron Syndrome are demonstrated to have increased insulin sensitivity and decreased T2DM incidence compared to their relatives, despite higher body fat content [126, 127]. Nevertheless, as shown in PAPE study, PEGV cannot mitigate the adverse effect of PASI in glucose homeostasis, when the 2 medications are co-administered, indicating that PEGV has no effect in insulin secretion [128].

### Dopamine agonizts (DAs)

Dopamine agonists (DAs) activate dopamine receptors type 2 (DR2), which may be expressed in somatotroph adenomas. DR2 are also expressed in pancreatic islets and adipocytes [129, 130]. Contrary to SRLS, the effect of DAs in glucose metabolism is beneficial [129]. In particular, bromocriptine improves glucose tolerance [131–133] and decreases insulin [131, 133] and glucagon [132] levels in patients with acromegaly [133]. Similarly, the addition of cabergoline to PEGV decreases the postprandial glucose rise [134].

Outside the concept of acromegaly, bromocriptine has been shown to decrease HbA1c, FPG, and mean glucose level during OGTT, in parallel with enhancing insulin sensitivity in “wild-type”, obese T2DM patients [135] and has been FDA-approved as an anti-diabetic medication [129]. This suggests that the anti-diabetic properties of DAs might be independent from their GH-suppressive effects. The underlying mechanisms may involve decrease in glucagon [132], direct effects in adipocytes and inhibition of prolactin (PRL) [129].

## Treatment of secondary and PASI-induced DM in acromegaly

Considering its secondary nature, the most effective treatment of DM is remission of acromegaly itself. Indeed, surgical treatment results into diabetes remission [10], improvement of glycemic status [7] and insulin sensitivity [33, 35, 36]. However, surgical remission cannot be achieved in 50% of cases [2] and medical therapy or radiotherapy may be needed. In view of widely recognized guidelines, the presence of secondary DM should not be considered as a criterion to choose among the above adjacent therapies. Furthermore, according to a large retrospective study, the prevalence of DM in acromegaly remains higher than in the general population, several years after multimodal therapy [17]. Therefore, anti-diabetic medications may be needed in order to achieve satisfactory glycemic control.

Few studies have addressed the management of secondary DM in acromegaly and the mainstay of treatment is no different from the guidelines of “wild-type” T2DM [136, 137]. According to a clinical study of 70 patients with acromegaly and secondary DM, 15.7% of patients are controlled on diet, 65.7% of patients receive metformin as monotherapy or in combination with other oral anti-diabetic medications and 21.5% of patients are insulin-treated, with all exhibiting excellent glycemic control (mean HbA1c = 6.4%) and low prevalence of diabetic microangiopathy [138]. Furthermore, thiazolidinediones have been demonstrated to achieve optimal glycemic control and allow insulin discontinuation in 2 case reports [139, 140]. Moreover, a recently-published case series in 9 patients receiving sodium-glucose-co-transporter 2 inhibitors (SGLT2is) shows 1% decrease in HbA1c without adverse events [141]. There is only one reported case of DKA in a SGLT2i-treated, diabetic patient with acromegaly, in which acromegaly was not previously recognized and SGLT2i was prescribed for presumed “wild-type” T2DM [142]; given that active acromegaly is also a DKA precipitant, the use of SGLT2is should be restricted to patients with biochemical control [143].

Regarding PASI-induced DM, a recently-published, multicentre study reveals superior efficacy of incretin-based therapy (sitagliptin followed by liraglutide) in comparison to insulin, as second-line treatment after metformin in 249 randomized patients [144]. This is in line with the recognition of incretin phenomenon inhibition as the key pathophysiologic mechanism of PASI-induced hyperglycemia [121].

Apart from the glucose-lowering potential of anti-diabetic medications in secondary DM, some of them have been suggested to bear disease-modifying or pleiotropic effects. In particular, rosiglitazone is shown to drastically decrease GH and IGF-1 level in a case with persistent acromegaly post-transsphenoidal surgery [139]. Interestingly, peroxisome proliferator-activated receptor gamma (PPARγ) is abundantly expressed in somatotropinomas, prolactinomas and gonadotropinomas and thiazolidinediones are demonstrated to inhibit hormonal secretion and tumor cell growth in vivo and in vitro, via inducing G0-G1 cell-cycle arrest and apoptosis [145]. Interestingly, a similar case of pioglitzone-induced remission of primary aldosteronism attributed to PPARγ-mediated suppression of β-catenin pathway is also reported in the literature [146]. In addition, the use of metformin has been recently shown to lower the prevalence of colonic polyps [147]. Finally, SGLT2is have been also speculated to exert a disease-modifying effect by lowering insulin secretion and therefore possibly decreasing IGF-1; however, no effects in GH or IGF-1 level are reported in the only, so far, case series examining their effect in acromegaly patients [141].

Taking everything into account, there are no robust clinical data on efficacy and safety of anti-diabetic agents in acromegaly, except for the case of PASI-induced DM [144]. Therefore, treating secondary DM as per guidelines for “wild-type” T2DM [148], in consideration of prevailing glucose metabolism defect in each patient and possible favorable or adverse impact of anti-diabetic agents in acromegaly or acromegaly-related complications, seems to be a rational approach.

## Conclusions and future perspectives

The key and primary defect of glucose metabolism in acromegaly is insulin resistance, deriving principally from excessive lipolysis and altered fat distribution; this is reflected at the presence of intermuscular fat and attenuated yet dysfunctional adipose tissue.

**Fig. 1:**
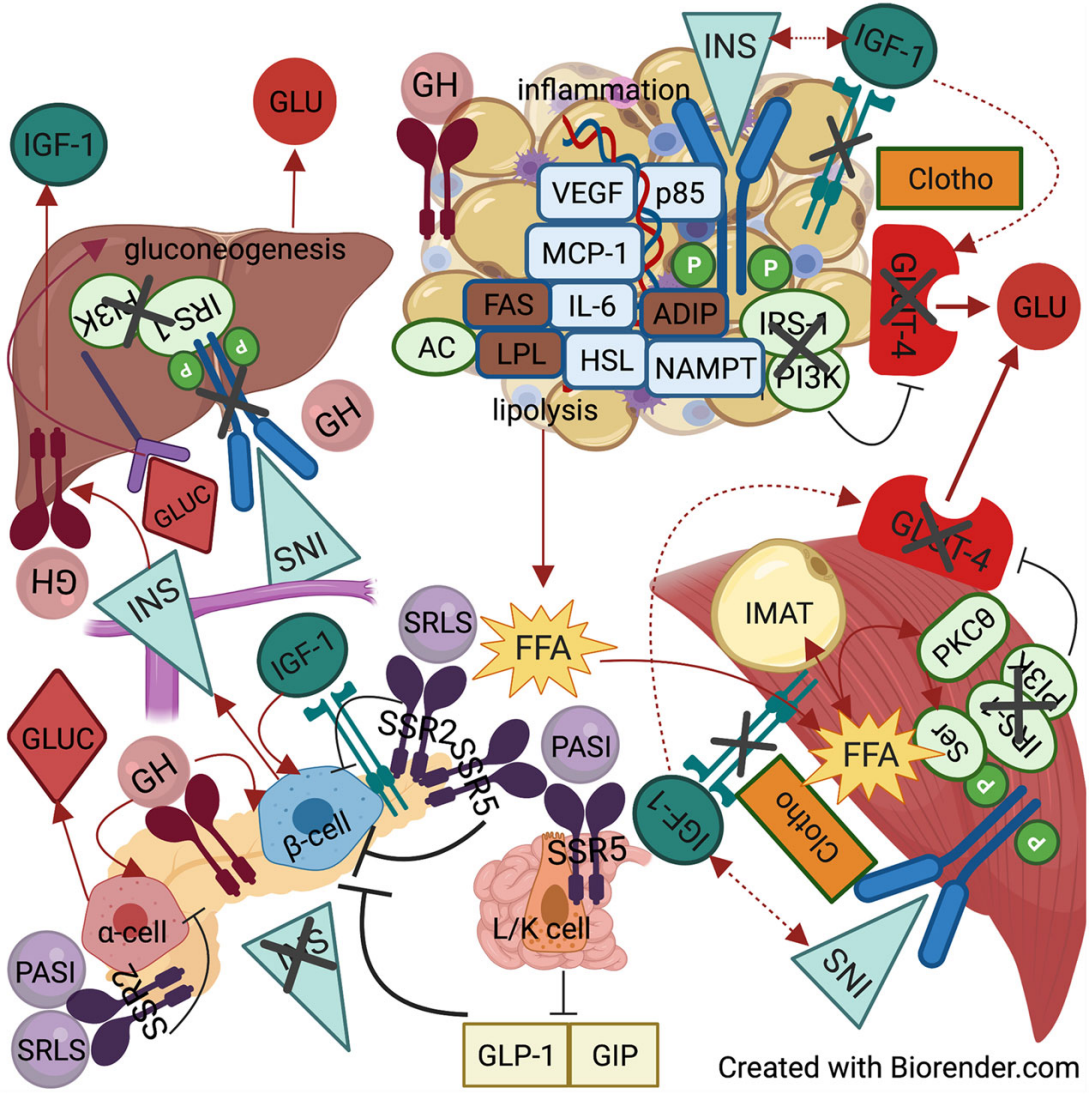
Pathophysiological model of secondary DM in acromegaly and the effects of SRLS and PASI in glucose metabolism. Cross-talk among muscle, liver, adipose tissue pancreas, and gut. In adipose tissue, growth hormone (GH) increases the expression of p85 subunit of phosphatidylinositol-3-kinase (PI3K), which is a negative regulator of the latter, leading to inhibition of IRS-1/PI3K pathway and ultimately, to decreased glucose transporter-4 (GLUT-4) translocation and glucose (GLU) uptake. GH also decreases adipokine (ADIP) expression, precipitating insulin resistance. In parallel, there is increased expression of hormone-sensitive lipase (HSL) and decreased expression of the lipogenic enzymes lipoprotein lipase (LPL) and fatty acid synthase (FAS), resulting into excessive lipolysis and free fatty acid (FFA) generation; lipolysis is further promoted by enhanced adenylate cyclase (AC) function. Finally, GH upregulates the expression of nicotinamide phosphorybosultransferase (NAMPT) and inflammatory cytokines, such as monocyte chemotactic protein 1 (MCP-1), vascular endothelial growth factor-A (VEGF-A) and interleukin-6 (IL-6), inducing a state of inflammation. Circulating FFAs are shifted in muscle, where they form intermuscular adipose tissue (IMAT) and activate protein kinase C theta (PKCθ). In turn, PKCθ favors serine (Ser) over tyrosine phosphorylation of insulin receptor substrate 1 (IRS-1), resulting in inhibition of PI3K activity and subsequent decrease in GLUT-4 translocation and GLU uptake. Both adipose tissue and muscle express IGF-1 receptors, which may interact with insulin receptors forming hybrid receptors; however, they do not manage to compensate for insulin resistance via increasing GLUT-4 translocation, due to IGF-1 receptor resistance induced by IGF-1 excess, hyperinsulinemia and increased level of Clotho. In pancreas, GH stimulates insulin (INS) and glucagon (GLUC) secretion, while IGF-1 acts synergistically in stimulating INS secretion. As a result, great amounts of INS are secreted in portal vein, leading to increased responsiveness of liver GH receptor and IGF-1 production. Meanwhile, maximal basal stimulation of liver insulin receptors results into their desensitization, downregulation, and eventually, to increased gluconeogenesis. This is possibly exacerbated by hyperglucagonemia. First-generation somatostatin receptor ligands (SRLS) inhibit INS secretion by activating somatostatin receptor subtype 2 (SSR2) in beta pancreatic cells (β-cells). Pasireotide (PASI) inhibits INS secretion by activating somatostatin receptor subtype 5 (SSR5) in β-cells as well as in K and L enteroendocrine cells in gut, with the latter leading to decreased incretin secretion. Considering the latter in conjuction with greater abundance of β-cells in SSR5 than SSR2, justifies the greater insulin inhibitory effect of PASI in comparison to SRLS (represented by thicker inhibitory line). Both PASI and SRLS inhibit glucagon secretion by activating SSR2 in alpha cells. GH-responsive genes are represented by parallelograms, colored light blue if upregulated and brown if downregulated. Intracellular proteins are represented by light green oval shape. GH is represented by pink oval shape, IGF-1 by cyan blue oval shape, SRLS and PASI by purple oval shape, GLU by red oval shape, GLUC by diamond red shape FFA by yellow star-like shape, INS by light blue triangles, and Clotho by orange parallelograms. The occurring stimulatory or inhibitory effects are represented by solid arrow and inhibitor lines; dashed arrow lines are used to describe the effects of IGF-1 which are impeded owing to IGF-1 receptor resistance. AC adenylate cyclase, ADIP adiponectin, FAS fatty acid synthase, FFA free fatty acid, GH growth hormone, GIP glucose-dependent insulinotropic polypeptide, GLP-1 glucagon-like peptide 1, GLU glucose, GLUC glucagon, GLUT-4 glucose transporter-4, HSL hormone-sensitive lipase, IGF-1 insulin-like growth factor 1, IL-6 interleukin 6, IMAT intermuscular adipose tissue, INS insulin, IRS-1 insulin receptor substrate 1, K cell K enteroendocrine cell, L cell L enteroendocrine cell, LPL lipoprotein lipase, MCP-1 monocyte chemotactic protein 1, p85 p85 subunit of PI3K, P phosphorylation, PASI Pasireotide, PI3K phosphatidylinositol-3-kinase, PKCθ protein kinase C theta, Ser serine, SSR2 somatostatin receptor subtype 2, SSR5 somatostatin receptor subtype 5, VEGF vascular endothelial growth factor, α-cell alpha pancreatic cell, β-cell beta pancreatic cell

The actions of GH and IGF-1 are antagonistic; GH induces insulin resistance while IGF-1 enhances insulin sensitivity. Based on the net effect in glucose metabolism, it is evident that the diabetogenic effect of GH supersedes the insulin-sensitizing effect of IGF-1 in target tissues. This might be attributed to higher glucometabolic potency of GH, IGF-1 resistance, or both Fig. 1. It would be interesting to explore how IGF-1R becomes selectively resistant as per its metabolic but not mitogenic functions in future studies in vitro. It should be noted that most of our knowledge regarding GH and IGF-1 effects is based on non-acromegalic models of acute GH or IGF-1 administration. In addition, the role of hybrid receptors in acromegaly has not been explored. Therefore, more basic research in animal models or tissues from patients with acromegaly is required to unravel the molecular basis of insulin resistance. Furthermore, future clinical studies could elaborate a possible pathophysiologic contribution of concomitant hyperprolactinemia and even reveal an additional benefit from DAs in this subset of patients.

Insulin resistance is initially compensated by increased insulin secretion, triggered synergistically by GH and IGF-1, as well as by insulin resistance Fig. 1. IGT and DM occur upon beta cell exhaustion due to gluco-lipo-toxicity, similarly to “wild-type” T2DM. Unlike insulin resistance, which is amenable upon acromegaly remission or control, impaired insulin secretion is hard to reverse and might lead to DM persistence after acromegaly treatment.

Medications used in the treatment of acromegaly may affect glucose homeostasis per se, with SRLS, especially PASI, inhibiting insulin secretion Fig. 1 and PEGV and DAs improving insulin sensitivity. Emerging evidence indicates that PASI-induced DM is a distinct pathophysiologic entity arising mainly from incretin phenomenon inhibition; the possible contribution of SSR1 and SSR3 activation to this phenomenon warrants further investigation in vitro Fig. 1, Table 1.

**Table 1:** Hyperglycemia in acromegaly

Causes of hyperglycemia	Pathophysiologic mechanisms			Glycemic phenotype	Suggested management
**Insulin resistance**	**Muscle** (GH prevails over IGF-1)	↑ IMAT + FFA uptake→ ↑ PKCθ → ↓ IRS-1/PI3K activity → ↓ glucose uptake		**NGT** (insulin resistance + ↑ ↑ insulin secretion)	Metformin/Pioglitazone
		↓ glucose oxidation			
	**Adipose tissue** (GH prevails over IGF-1)	↓ lipogenesis, ↑ lipolysis → ↓ TAT/SAT		**IGF/IGT** (insulin resistance + ↓ insulin secretion)	
		↑p85 → ↓ PI3K activity → ↓ glucose uptake			
		↓ adiponectin & leptin			
		↑ NAMPT → ↓ glucolysis			
		Inflammation (via ↑ inflammatory cytokines & adipokines)			
	**Liver** (GH only)	insulin and GH overstimulation → IRS-1/PI3K desensitization + IR downregulation → ↑ gluconeogenesis		**DM** (insulin resistance + ↓ ↓ insulin secretion)	Metformin/Pioglitazone ↓ SGLT2is*/ Incretin secretagogues ↓ Insulin
**Impaired insulin secretion**	**Beta cell exhaustion**	gluco-lipo-toxicity GIP resistance			
	**Pasireotide**	SSR5, SSR1?, SSR3?activation	Beta cells → ↓INS	**IGF/IGT** (↓ insulin secretion ± insulin resistance)	Metformin/Pioglitazone (in the presence of insulin resistance)
			K and L cells → ↓ GIP, GLP-1	**DM** (↓↓ insulin secretion ± insulin resistance)	Metformin ↓ Incretin secretagogues ± SGLT2is*/pioglitazone ↓ Insulin

The table is divided in 2 main subsections (raws), corresponding to the 2 main causes of hyperglycemia, insulin resistance and impaired insulin secretion. For each of the two main causes, the corresponding pathophysiologic mechanisms, glycemic phenotype and suggested management is presented (columns). The pathophysiological mechanisms of insulin resistance are subclassified as per the 3 insulin-sensitive tissues and impaired insulin secretion is also etiologically subclassified into beta cell exhaustion- and pasireotide-related. The suggested management matches each clinical phenotype and the principal pathophysiologic mechanism. The presence of cardiovascular disease, heart failure and chronic kidney disease has not been considered in suggested management here, but, if present, the current guidelines for T2DM with glucagon-like peptide 1 receptor agonizts (GLP-1RA) or sodium-glucose co-transporter 2 inhibitors (SGLT2i) should be followed.* only in cases of controlled acromegaly, ↑ increase, ↑↑ big increase ↓ decrease, ↓↓ big decrease, → results into, + plus, ± plus or minus, DM diabetes mellitus, FFA free fatty acid, GH growth hormone, GIP glucose-dependent insulinotropic polypeptide, GLP-1 glucagon-like peptide 1, IMAT intrermuscular adipose tissue, IR insulin receptor, IRS-1 insulin receptor substrate 1, NAMPT nicotinamide phosphorybosultransferase (also known as visfatin), PI3K phosphatidylinositol-3-kinase, PKCθ protein kinase C theta, SAT subcutanenous adipose tissue, SGLT2is sodium-glucose co-transporter 2 inhibitors, SSR1 somatostatin receptor subtype 1, SSR3 somatostatin receptor subtype 3, SSR5 somatostatin receptor subtype 5, TAT total adipose tissue [5, 28, 33–36, 38, 39, 41–43, 45–48, 50, 51, 60–63, 65, 66, 68, 69, 72, 84, 94, 114–119, 121, 139–141, 144, 148]

Keeping in view of the literature data, it would be meaningful to attempt a pathophysiologic classification of patients with acromegaly and insulin resistance and/or impaired glucose homeostasis as: (i) patients with insulin resistance and NGT, (ii) patients with concomitant insulin resistance and impaired insulin secretion (IGT,DM), (iii) patients with SRLS/PASI-induced DM/IGT with sub classification according to pre-existing IGT/DM or new-onset IGT/DM (Table 1).

Secondary DM in acromegaly is mild and easily controlled by diet or pharmacotherapy. However, it has a discordantly huge impact in morbidity and mortality, arising mainly from cardiovascular disease and neoplasia. In the light of the GH-IGF-1/insulin interplay, we are led to believe that hyperinsulinemia might be more detrimental than hyperglycemia. In conjunction with limited but fascinating data pointing towards disease-modifying effects of metformin and pioglitazone, it is worth to explore if treatment with insulin sensitizers could confer clinical benefits in patients with insulin resistance and NGT in prospective, interventional, cohort trials. Similarly, the potential of SGLT2is to prevent cardiovascular and/or other acromegaly complications in appropriately selected patients deserves further investigation.

In conclusion, our perspective towards secondary DM in acromegaly should broaden beyond glycemic control, in order to embrace the impact of its presence and management to acromegaly per se. Pathophysiologic classification of DM in acromegaly and accordingly guided management seems to be the only way forward.

## Supplementary Information

Publication License Feb-16-2023

## References

[1] Fleseriu M, Langlois F, Lim DST, Varlamov EV, Melmed S (2022). Acromegaly: pathogenesis, diagnosis, and management. Lancet Diabetes Endocrinol..

[2] A. Colao, L.F.S. Grasso, A. Giustina, S. Melmed, P. Chanson, A.M. Pereira, R. Pivonello, Acromegaly. Nat. Rev. Dis. Primers. **5**, (2019). 10.1038/s41572-019-0071-6 10.1038/s41572-019-0071-6 30899019

[3] Darragh JH, Shaw WM (1951). Acromegaly and Diabetes. Canad. Med. Assoc. J..

[4] Gordon DA, Hill FM, Erzin C (1962). Acromegaly: A Review of 100 Cases. Canad. Med. Assoc. J..

[5] Alexopoulou O, Bex M, Kamenicky P, Mvoula AB, Chanson P, Maiter D (2014). Prevalence and risk factors of impaired glucose tolerance and diabetes mellitus at diagnosis of acromegaly: A study in 148 patients. Pituitary.

[6] Dreval AV, Trigolosova IV, Misnikova IV, Kovalyova YA, Tishenina RS, Barsukov IA, Vinogradova AV, Wolffenbuttel BHR (2014). Prevalence of diabetes mellitus in patients with acromegaly. Endocr. Connect.

[7] Li YL, Zhang S, Guo XP, Gao L, Lian W, Yao Y, Deng K, Wang RZ, Xing B (2019). Correlation analysis between short-term insulin-like growth factor-I and glucose intolerance status after transsphenoidal adenomectomy in acromegalic patients: A large retrospective study from a single center in China. Arch. Endocrinol. Metab..

[8] Kreze A, Kreze-Spirova E, Mikulecky M (2001). Risk factors for glucose intolerance in acromegaly. Braz. J. Med. Biol. Res..

[9] Hye Park K, Jig Lee E, Hyeon Seo G, Ryong Ku C (2020). Risk for acromegaly-related comorbidities by sex in Korean acromegaly. J. Clin. Endocrinol. Metab..

[10] Berkmann S, Brun J, Schuetz P, Christ E, Mariani L, Mueller B (2021). Prevalence and outcome of comorbidities associated with acromegaly. Acta Neurochir..

[11] Fieffe S, Morange I, Petrossians P, Chanson P, Rohmer V, Cortet C, Borson-Chazot F, Brue T, Delemer B, Sonnet E, Cazabat L, Hieronimus S, Gaillard R, Enjalbert A, Raingeard I, Dupuy O, Beckers A (2011). Diabetes in acromegaly, prevalence, risk factors, and evolution: Data from the French Acromegaly Registry. Eur. J. Endocrinol..

[12] Gatto F, Trifirò G, Lapi F, Cocchiara F, Campana C, Dell’Aquila C, Ferrajolo C, Arvigo M, Cricelli C, Giusti M, Ferone D (2018). Epidemiology of acromegaly in Italy: analysis from a large longitudinal primary care database. Endocrine.

[13] Vallette S, Ezzat S, Chik C, Ur E, Imran SA, van Uum S, Rivera J, Caspar-Bell G, Serri O (2013). Emerging trends in the diagnosis and treatment of acromegaly in Canada. Clin. Endocrinol..

[14] AlMalki MH, Ahmad MM, Buhary BM, Aljawair R, Alyamani A, Alhozali A, Alshahrani A, Alzahrani S, Nasser T, Alzahrani W, Raef H, Aldawish M, Elkhzaimy A (2020). Clinical features and therapeutic outcomes of patients with acromegaly in Saudi Arabia: a retrospective analysis. Hormones.

[15] Lesén E, Granfeldt D, Houchard A, Dinet J, Berthon A, Olsson DS, Björholt I, Johannsson G (2017). Comorbidities, treatment patterns and cost-of-illness of acromegaly in Sweden: A register-linkage population-based study. Eur. J. Endocrinol..

[16] Fukuda I, Hizuka N, Muraoka T, Kurimoto M, Yamakado Y, Takano K, Ichihara A (2014). Clinical features and therapeutic outcomes of acromegaly during the recent 10 years in a single institution in Japan. Pituitary.

[17] González B, Vargas G, de Los Monteros ALE, Mendoza V, Mercado M (2018). Persistence of Diabetes and Hypertension after Multimodal Treatment of Acromegaly. J. Clin. Endocrinol. Metab..

[18] Petrossians P, Daly AF, Natchev E, Maione L, Blijdorp K, Sahnoun-Fathallah M, Auriemma R, Diallo AM, Hulting AL, Ferone D, Hana V, Filipponi S, Sievers C, Nogueira C, Fajardo-Montañana C, Carvalho D, Hana V, Stalla GK, Jaffrain-Réa ML, Delemer B, Colao A, Brue T, Neggers SJCMM, Zacharieva S, Chanson P, Beckers A (2017). Acromegaly at diagnosis in 3173 patients from the Liège Acromegaly Survey (LAS) Database. Endocr. Relat. Cancer.

[19] Dal J, Feldt-Rasmussen U, Andersen M, Kristensen L, Laurberg P, Pedersen L, Dekkers OM, Sørensen HT, Jørgensen JOL (2016). Acromegaly incidence, prevalence, complications and long-term prognosis: A nationwide cohort study. Eur. J. Endocrinol..

[20] Rosario PW (2011). Frequency of acromegaly in adults with diabetes or glucose intolerance and estimated prevalence in the general population. Pituitary.

[21] Suda K, Fukuoka H, Iguchi K, Hirota Y, Nishizawa H, Bando H, Matsumoto R, Takahachi M, Sakaguchi K, Takahachi Y (2015). The prevalence of acromegaly in hospitilized patients with type 2 diabetes. Endocr. J..

[22] American Diabetes Association: Diagnosis and classification of diabetes mellitus. Diabetes Care. **37**, (2014). 10.2337/dc14-S081

[23] Yun SJ, Lee JK, Park SY, Chin SO (2021). Descriptive Epidemiology and Survival Analysis of Acromegaly in Korea. J. Korean Med. Sci..

[24] Colao A, Grasso LFS, di Cera M, Thompson-Leduc P, Cheng WY, Cheung HC, Duh MS, Neary MP, Pedroncelli AM, Maamari R, Pivonello R (2020). Association between biochemical control and comorbidities in patients with acromegaly: an Italian longitudinal retrospective chart review study. J. Endocrinol. Investig..

[25] S. Cheng, k. Gomez, O. Serri, C. Chik, S. Ezzat, The role of diabetes in acromegaly associated neoplasia. PLoS ONE. **10**, (2015). 10.1371/journal.pone.0127276 10.1371/journal.pone.0127276 PMC4440645 25996963

[26] Niculescu D, Purice M, Coculescu M (2013). Insulin-like growth factor-I correlates more closely than growth hormone with insulin resistance and glucose intolerance in patients with acromegaly. Pituitary.

[27] Esposito D, Olsson DS, Franzén S, Miftaraj M, Nåtman J, Gudbjörnsdottir S, Johannsson G (2022). Effect of Diabetes on Morbidity and Mortality in Patients With Acromegaly. J. Clin. Endocrinol. Metab..

[28] Frara S, Maffezzoni F, Mazziotti G, Giustina A (2016). Current and Emerging Aspects of Diabetes Mellitus in Acromegaly. Trends Endocrinol. Metabol..

[29] Nemes A, Kormányos Á, Domsik P, Kalapos A, Gyenes N, Lengyel C, Valkusz Z (2021). Diabetes mellitus deteriorates left ventricular deformation in acromegaly-analysis from the three-dimensional speckle-tracking echocardiographic MAGYAR-Path study. Quant. Imaging Med. Surg..

[30] Mazziotti G, Gola M, Bianchi A, Porcelli T, Giampietro A, Cimino V, Doga M, Gazzaruso C, de Marinis L, Giustina A (2011). Influence of diabetes mellitus on vertebral fractures in men with acromegaly. Endocrine.

[31] H.N. Jang, Y.H. Kim, J.H. Kim, Diabetes Mellitus Predicts Weight Gain After Surgery in Patients with Acromegaly. Front. Endocrinol. 13, (2022). 10.3389/fendo.2022.854931 10.3389/fendo.2022.854931 PMC8959539 35355553

[32] Tseng FY, Chen ST, Chen JF, Huang TS, Lin J, der, Wang PW, Huey-Herng Sheu W, Chang TC (2019). Correlations of clinical parameters with quality of life in patients with acromegaly: Taiwan Acromegaly Registry. J. Formos. Med. Assoc..

[33] Biagetti B, Aulinas A, Casteras A, Pérez-Hoyos S, Simó R (2021). HOMA-IR in acromegaly: a systematic review and meta-analysis. Pituitary.

[34] D.A. Niculescu, R. Dusceac, A. Caragheorgheopol, N. Popescu, C. Poiana, Disposition Index in Active Acromegaly. Front. Endocrinol. **10**, (2019). 10.3389/fendo.2019.00637 10.3389/fendo.2019.00637 PMC6759813 31620090

[35] Wasada T, Aoki K (1997). Assessment of Insulin Resistance in Acromegaly Associated with Diabetes Mellitus before and after Transsphenoidal Adenomectomy. Endocr. J..

[36] Mori K, Iwasaki Y, Kawasaki-Ogita Y, Honjo S, Hamamoto Y, Tatsuoka H, Fujimoto K, Ikeda H, Wada Y, Takahashi Y, Takahashi J, Koshiyama H (2013). Improvement of insulin resistance following transsphenoidal surgery in patients with acromegaly: Correlation with serum IGF-I levels. J. Endocrinol. Investig..

[37] A. Ciresi, M.C. Amato, R. Pivonello, E. Nazzari, L.F. Grasso, F. Minuto, D. Ferone, A. Colao, C. Giordano, The metabolic profile in active acromegaly is gender-specific. J. Clin. Endocrinol. Metabol. **98**, (2013). 10.1210/jc.2012-2896 10.1210/jc.2012-2896 23162101

[38] Moøller N, Joørgensen JOL (2009). Effects of growth hormone on glucose, lipid, and protein metabolism in human subjects. Endocr. Rev..

[39] Nielsen S, Møller N, Christiansen JS, Jørgensen JO, Otto Lunde Jør-gensen J (2001). Pharmacological Antilipolysis Restores Insulin Sensitivity During Growth Hormone Exposure. Diabetes.

[40] P.J. Randle, P.B. Garland, C.N. Hales, E.A. Newsholme, The Glucose Fatty-Acid Cycle. Its Role in Insulin Sensitivity and the Metabolic Disturbances of Diabetes Mellitus. Lancet **1**, 785–789 (1963) 10.1016/s0140-6736(63)91500-9 13990765

[41] Shulman GI (2014). Ectopic Fat in Insulin Resistance, Dyslipidemia, and Cardiometabolic Disease. N. Engl. J. Med.

[42] Freda PU, Shen W, Heymsfield SB, Reyes-Vidal CM, Geer EB, Bruce JN, Gallagher D (2008). Lower visceral and subcutaneous but higher intermuscular adipose tissue depots in patients with growth hormone and insulin-like growth factor I excess due to acromegaly. J. Clin. Endocrinol. Metab..

[43] Reyes-Vidal CM, Mojahed H, Shen W, Jin Z, Arias-Mendoza F, Fernandez JC, Gallagher D, Bruce JN, Post KD, Freda PU (2015). Adipose tissue redistribution and ectopic lipid deposition in active acromegaly and effects of surgical treatment. J. Clin. Endocrinol. Metab..

[44] Giustina A, Bresciani E, Tassi C, Girelli A, Valentini U (1994). Effect of Pyridostigmine on the Growth Hormone Response to Growth Hormone-Releasing Hormone in Lean and Obese Type II Diabetic Patients. Metabolism.

[45] Fineberg SE, Merimee TJ (1974). Acute Metabolic Effects of Human Growth Hormone. Diabetes.

[46] D. Rabinowitz, G.A. Klassen, K.L. Zierler, Effect of Human Growth Hormone on Muscle and Adipose Tissue Metabolism in the Forearm of Man*. J. Clin. Investig. **44**, 51–61 (1965) 10.1172/JCI105126 PMC442018 14254256

[47] Griffin ME, Marcucci MJ, Cline GW, Bell K, Barucci N, Lee D, Goodyear LJ, Kraegen EW, White MF, Shulman GI (1999). Free Fatty Acid-Induced Insulin Resistance Is Associated With Activation of Protein Kinase C θ and Alterations in the Insulin Signaling Cascade. Diabetes.

[48] Dresner A, Laurent D, Marcucci M, Griffin ME, Dufour S, Cline GW, Slezak LA, Andersen DK, Hundal RS, Rothman DL, Falk Petersen K, Shulman GI (1999). Effects of free fatty acids on glucose transport and IRS-1-associated phosphatidylinositol 3-kinase activity. J. Clin. Investig..

[49] Jessen N, Djurhuus CB, Jørgensen JOL, Jensen LS, Møller N, Lund S, Schmitz O (2005). Evidence against a role for insulin-signaling proteins PI 3-kinase and Akt in insulin resistance in human skeletal muscle induced by short-term GH infusion. Am. J. Physiol. Endocrinol. Metab..

[50] Moller N, Schmitz O, Jorgensen JL, Astrup J, Bak JF, Christensen SE, George K, Alberti MM: (1992). Basal-and Insulin-Stimulated Substrate Metabolism in Patients with Active Acromegaly Before and After Adenomectomy*. J. Clin. Endocrinol. Metab..

[51] Krag MB, Gormsen LC, Guo Z, Christiansen JS, Jensen MD, Nielsen S, L Jørgensen JO (2007). Growth hormone-induced insulin resistance is associated with increased intramyocellular triglyceride content but unaltered VLDL-triglyceride kinetics. Am. J. Physiol. Endocrinol. Metab..

[52] J. Szendroedi, E. Zwettler, A.I. Schmid, M. Chmelik, G. Pacini, G. Kacerovsky, G. Smekal, P. Nowotny, O. Wagner, C. Schnack, G. Schernthaner, K. Klaushofer, M. Roden, Reduced basal ATP synthetic flux of skeletal muscle in patients with previous acromegaly. PLoS ONE. **3**, (2008). 10.1371/journal.pone.0003958 10.1371/journal.pone.0003958 PMC2599885 19093000

[53] Goodpaster BH, Thaete FL, Kelley DE (2000). Thigh adipose tissue distribution is associated with insulin resistance in obesity and in type 2 diabetes mellitus. Am. J. Clin. Nutr..

[54] Albu JB, Kovera AJ, Allen L, Wainwright M, Berk E, Raja-Khan N, Janumala I, Burkey B, Heshka S, Gallagher D (2005). Independent association of insulin resistance with larger amounts of intermuscular adipose tissue and a greater acute insulin response to glucose in African American than in white nondiabetic women. Am. J. Clin. Nutr..

[55] Albu JB, Kenya S, He Q, Wainwright M, Berk ES, Heshka S, Kotler DP, Engelson ES (2007). Independent associations of insulin resistance with high whole-body intermuscular and low leg subcutaneous adipose tissue distribution in obese HIV-infected women. Am. J. Clin. Nutr..

[56] Ottosson M, Vikman-Adolfsson K, Enerback S, Elander A, Bjorntorp P, Eden S (1995). Growth Hormone Inhibits Lipoprotein Lipase Activity in Human Adipose Tissue. J. Clin. Endocrinol. Metab..

[57] Yin D, Clarke SD, Peters JL, Etherton TD (1998). Somatotropin-dependent decrease in fatty acid synthase mRNA abundance in 3T3-F442A adipocytes is the result of a decrease in both gene transcription and mRNA stability. Biochem. J..

[58] Yang S, Mulder H, Holm C, Edén S (2004). Effects of growth hormone on the function of β-adrenoceptor subtypes in rat adipocytes. Obes. Res.

[59] Beauville M, Harant I, Crampes F, Riviere D, Tauber MT, Tauber JP, Garrigues M (1992). Effect of long-term rhGH administration in GH-deficient adults on fat cell epinephrine response. Am. J. Physiol. Endocrinol. Metabol..

[60] del Rincon JP, Iida K, Gaylinn BD, McCurdy CE, Leitner JW, Barbour LA, Kopchick JJ, Friedman JE, Draznin B, Thorner MO (2007). Growth Hormone Regulation of p85α Expression and Phosphoinositide 3-Kinase Activity in Adipose tissue: Mechanism for Growth Hormone-Mediated Insulin Resistance. Diabetes.

[61] Bolanowski M, Milewicz A, Bidzińska B, Jędrzejuk D, Daroszewski De J, Mikulski E (2002). Serum leptin levels in acromegaly-a significant role for adipose tissue and fasting insulin/glucose ratio. Med. Sci. Monit..

[62] Damjanovic SS, Petakov MS, Raicevic S, Micic D, Marinkovic J, Dieguez C, Casanueva FF, Popovic V (2000). Serum Leptin Levels in Patients with Acromegaly before and after Correction of Hypersomatotropism by Trans-Sphenoidal Surgery. J. Clin. Endocrinol. Metab..

[63] Wiesli P, Bernays R, Brändle M, Zwimpfer C, Seiler H, Zapf J, A. Spinas G, Schmid C (2005). Effect of pituitary surgery in patients with acromegaly on adiponectin serum concentrations and alanine aminotransferase activity. Clin. Chim. Acta.

[64] S. Gariballa, J. Alkaabi, J. Yasin, A. al Essa, Total adiponectin in overweight and obese subjects and its response to visceral fat loss. BMC Endocr. Disord. **19**, (2019). 10.1186/s12902-019-0386-z 10.1186/s12902-019-0386-z PMC6545728 31159801

[65] White UA, Maier J, Zhao P, Richard AJ, Stephens JM (2016). The modulation of adiponectin by STAT5-activating hormones. Am. J. Physiol. Endocrinol. Metab..

[66] Olarescu NC, Ueland T, Lekva T, Dahl TB, Halvorsen B, Aukrust P, Bollerslev J (2012). Adipocytes as a source of increased circulating levels of nicotinamide phosphoribosyltransferase/visfatin in active acromegaly. J. Clin. Endocrinol. Metab..

[67] Ciresi A, Amato MC, Pizzolanti G, Giordano C (2015). Serum visfatin levels in acromegaly: Correlation with disease activity and metabolic alterations. Growth Horm. IGF Res.

[68] Olarescu NC, Bollerslev J (2016). The Impact of Adipose Tissue on Insulin Resistance in Acromegaly. Trends Endocrinol. Metab..

[69] Dominici FP, Cifone D, Bartke A, Turyn D (1999). Loss of sensitivity to insulin at early events of the insulin signaling pathway in the liver of growth hormone-transgenic mice. J. Endocrinol..

[70] Tai T-Y, Pek S (1976). Direct Stimulation by Growth Hormone of Glucagon and Insulin Release from Isolated Rat Pancreas. Endocrinology.

[71] Sirek A, Vranic M, Sirek V, Vigas M, Policova Z (1979). Effect of growth hormone on acute glucagon and insulin release. Am. J. Physiol..

[72] Trimble ER, Brew Atkinson A, Buchanan KD, Hadden DR (1980). Plasma Glucagon and Insulin Concentrations in Acromegaly. J. Clin. Endocrinol. Metab..

[73] Clemmons DR (2002). Roles of Insulin-like Growth Factor-I and Growth Hormone in Mediating Insulin Resistance in Acromegaly. Pituitary.

[74] Moses AC, Young SCJ, Morrow LA, O’brien M, Clemmons DR (1996). Recombinant Human Insulin-like Growth Factor I Increases Insulin Sensitivity and Improves Glycemic Control in Type II Diabetes. Diabetes.

[75] Jin Chan S, Steiner DF (2000). Insulin Through the Ages: Phylogeny of a Growth Promoting and Metabolic Regulatory Hormone. Am. Zool..

[76] Ullrich A, Gray A, Tam AW, Yang-Feng T, Tsubokawa M, Collins C, Henzel W, le Bon T, Kathuria S, Chen E, Jacobs S, Francke U, Ramachandran J, Fujita-Yamaguchi Y (1986). Insulin-like growth factor I receptor primary structure: comparison with insulin receptor suggests structural determinants that define functional specificity. EMBO J..

[77] Dohm GL, Elton CW, Raju MS, Mooney ND, Dimarchi R, Pories WJ, Flickinger EG, Atkinson SM, Caro JF (1990). jGF-l-Stimulated Glucose Transport in Human Skeletal Muscle and IGF-I Resistance in Obesity and NIDDM. Diabetes.

[78] Sinha MK, Buchanan C, Leggett N, Martin L, Khazanie PG, Dimarchi R, Pories WJ, Caro JF (1989). Mechanism of IGF-I-Stimulated Glucose Transport in Human Adipocytes Demonstration of Specific IGF-I Receptors Not Involved in Stimulation of Glucose Transport. Diabetes.

[79] Kineman RD, del Rio-Moreno M, Sarmento-Cabral A (2018). 40 years of IGF1: Understanding the tissue-specific roles of IGF1/IGF1R in regulating metabolism using the Cre/loxP system. J. Mol. Endocrinol..

[80] Federici M, Porzio O, Lauro D, Borboni P, Giovannone B, Zucaro L, Letizia Hribal M, Sesti G (1998). Increased Abundance of Insulin/Insulin-Like Growth Factor-I Hybrid Receptors in Skeletal Muscle of Obese Subjects Is Correlated with In Vivo Insulin Sensitivity.*. J. Clin. Endocrinol. Metab..

[81] Federici M, Zucaro L, Porzio O, Massoud R, Borboni P, Lauro D, Sesti G (1996). Increased Expression of Insulin/Insulin-like Growth Factor-I Hybrid Receptors in Skeletal Muscle of Noninsulin-dependent Diabetes Mellitus Subjects. J. Clin. Investig.

[82] Federici M, Porzio O, Zucaro L, Giovannone B, Borboni P, Marini MA, Lauro D, Sesti G (1997). Increased abundance of insulin/IGF-I hybrid receptors in adipose tissue from NIDDM patients. Mol. Cell. Endocrinol..

[83] Halleux CM, Takahashi M, Delporte ML, Detry R, Funahashi T, Matsuzawa Y, Brichard SM (2001). Secretion of adiponectin and regulation of apM1 gene expression in human visceral adipose tissue. Biochem. Biophys. Res. Commun..

[84] Fukuoka H, Takahashi Y, Iida K, Kudo T, Nishizawa H, Imanaka M, Takeno R, Iguchi G, Takahashi K, Okimura Y, Kaji H, Chihara K (2008). Low serum IGF-I/GH ratio is associated with abnormal glucose tolerance in acromegaly. Horm. Res.

[85] Yakar S, Liu J-L, Fernandez AM, Wu Y, Schally AV, Frystyk J, Chernausek SD, Mejia W, le Roith D (2001). Liver-Specific igf-1 Gene Deletion Leads to Muscle Insulin Insensitivity. Diabetes.

[86] Yakar S, Setser J, Zhao H, Stannard B, Haluzik M, Glatt V, Bouxsein ML, Kopchick JJ, LeRoith D (2004). Inhibition of growth hormone action improves insulin sensitivity in liver IGF-1-deficient mice. J. Clin. Invest.

[87] J.A.M.J.L. Janssen, Mechanisms of putative IGF-I receptor resistance in active acromegaly. Growth Horm. IGF Res. **52**, (2020). 10.1016/j.ghir.2020.101319 10.1016/j.ghir.2020.101319 32339897

[88] Fineberg SE, Merimee TJ, Rabinowitz D, Edgar PJ (1970). Insulin Secretion in Acromegaly. J. Clin. Endocrinol..

[89] Xuan S, Kitamura T, Nakae J, Politi K, Kido Y, Fisher PE, Morroni M, Cinti S, White MF, Herrera PL, Accili D, Efstratiadis A (2002). Defective insulin secretion in pancreatic β cells lacking type 1 IGF receptor. J. Clin. Invest.

[90] Wu Y, Liu C, Sun H, Vijayakumar A, Giglou PR, Qiao R, Oppenheimer J, Yakar S, Leroith D (2011). Growth hormone receptor regulates β cell hyperplasia and glucose-stimulated insulin secretion in obese mice. J. Clin. Invest.

[91] Nielsen JH, Galsgaard ED, Møldrup A, Friedrichsen N, Billestrup N, Hansen JA, Lee YC, Carlsson C (2001). Regulation of β-Cell Mass by Hormones and Growth Factors. Diabetes.

[92] Z. Wang, L. Gao, X. Guo, C. Feng, K. Deng, W. Lian, M. Feng, X. Bao, B. Xing, Preoperative Fasting C-Peptide Acts as a Promising Predictor of Improved Glucose Tolerance in Patients With Acromegaly After Transsphenoidal Surgery: A Retrospective Study of 64 Cases From a Large Pituitary Center in China. Front. Endocrinol. **10**, (2019). 10.3389/fendo.2019.00736 10.3389/fendo.2019.00736 PMC6838023 31736874

[93] He W, Yan L, Wang M, Li Q, He M, Ma Z, Ye Z, Zhang Q, Zhang Y, Qiao N, Lu Y, Ye H, Lu B, Shou X, Zhao Y, Li Y, Li S, Zhang Z, Shen M, Wang Y (2019). Surgical outcomes and predictors of glucose metabolism alterations for growth hormone-secreting pituitary adenomas: a hospital-based study of 151 cases. Endocrine.

[94] V.S. Shekhawat, S. Bhansali, P. Dutta, K.K. Mukherjee, K. Vaiphei, R. Kochhar, S.K. Sinha, N. Sachdeva, A.V. Kurpad, Bhat, K., Mudaliar, S., Bhansali, A. Glucose-dependent Insulinotropic Polypeptide (GIP) Resistance and β-cell Dysfunction Contribute to Hyperglycaemia in Acromegaly. Sci. Rep. 9, (2019). 10.1038/s41598-019-41887-7 10.1038/s41598-019-41887-7 PMC6449401 30948746

[95] Yoshida N, Goto H, Suzuki H, Nagasawa K, Takeshita A, Okubo M, Miyakawa M, Mori Y, Fukuhara N, Nishioka H, Yamada S, Takeuchi Y (2013). Ketoacidosis as the initial clinical condition in nine patients with acromegaly: A review of 860 cases at a single institute. Eur. J. Endocrinol..

[96] P. Palakawong, R. Arakaki, Diabetic Ketoacidosis in Acromegaly: A Case Report. Endocr. Pract. 1–15 (2012). 10.4158/ep12189.cr 10.4158/EP12189.CR 23186966

[97] Weiss J, Wood AJ, Zajac JD, Grossmann M, Andrikopoulos S, Ekinci EI (2017). Diabetic ketoacidosis in acromegaly; a rare complication precipitated by corticosteroid use. Diabetes Res. Clin. Pract..

[98] Singla M, Saini JK (2021). Diabetes Mellitus of Pituitary Origin: A Case Report. Eur. Endocrinol..

[99] S.I. Chisalita, J. Ludvigsson, Insulin-like growth factor-1 at diagnosis and during subsequent years in adolescents with type 1 diabetes. J Diabetes Res. (2018). 10.1155/2018/8623560 10.1155/2018/8623560 PMC5883934 29744370

[100] Suda K, Matsumoto R, Fukuoka H, Iguchi G, Hirota Y, Nishizawa H, Bando H, Yoshida K, Odake Y, Takahashi M, Sakaguchi K, Ogawa W, Takahashi Y (2016). The influence of type 2 diabetes on serum GH and IGF-I levels in hospitalized Japanese patients. Growth Horm. IGF Res.

[101] Frystyk J, Ritzel RA, Maubach J, Büsing M, Lück R, Klempnauer J, Schmiegel W, Nauck MA (2008). Comparison of pancreas-transplanted type 1 diabetic patients with portal-venous versus systemic-venous graft drainage: Impact on glucose regulatory hormones and the growth hormone/insulin-like growth factor-I axis. J. Clin. Endocrinol. Metab..

[102] D.R. Wijayaratne, M.H. Arambewela, C. Dalugama, C., Wijesundera, D., Somasundaram, N., Katulanda, P.: Acromegaly presenting with low insulin-like growth factor-1 levels and diabetes: A case report. J. Med. Case Rep. 9, (2015). 10.1186/s13256-015-0736-z 10.1186/s13256-015-0736-z PMC4627376 26514337

[103] Jun Lim D, Sang Kwon H, Hyoung Cho J, Hee Kim S, Hee Choi Y, Yoon KH, Yun Cha B, Woo Lee K, Young Son H, Koo Kang S (2007). Acromegaly Associated with Type 2 Diabetes Showing Normal IGF-1 Levels under Poorly Controlled Glycemia. Endocr. J..

[104] Droste M, Domberg J, Buchfelder M, Mann K, Schwanke A, Stalla G, Strasburger CJ (2014). Therapy of acromegalic patients exacerbated by concomitant type 2 diabetes requires higher pegvisomant doses to normalise IGF1 levels. Eur. J. Endocrinol..

[105] Kumar U, Sasi R, Suresh S, Patel A, Thangaraju M, Metrakos P, Patel SC, Patel YC (1999). Subtype-Selective Expression of the Five Somatostatin Receptors (hSSTR1-5) in Human Pancreatic Islet Cells A Quantitative Double-Label Immunohistochemical A n a l y s i s. Diabetes.

[106] Giustina A, Mazziotti G, Maffezzoni F, Amoroso V, Berruti A (2014). Investigational drugs targeting somatostatin receptors for treatment of acromegaly and neuroendocrine tumors. Exp. Opin. Investig. Drugs.

[107] Mazziotti G, Floriani I, Bonadonna S, Torri V, Chanson P, Giustina A (2009). Effects of somatostatin analogs on glucose homeostasis: A metaanalysis of acromegaly studies. J. Clin. Endocrinol. Metab..

[108] Sagvand BT, Khairi S, Haghshenas A, Swearingen B, Tritos NA, Miller KK, Klibanski A, Nachtigall LB (2016). Monotherapy with lanreotide depot for acromegaly: long-term clinical experience in a pituitary center. Pituitary.

[109] M. Shen, M. Wang, W. He, M. He, N. Qiao, Z. Ma, Z. Ye, Q. Zhang, Y. Zhang, Y. Yang, Y. Cai, Y. Abuduoreyimu, Y. Lu, B. Lu, X. Shou, Y. Wang, H. Ye, Y. Li, S. Li, Y. Zhao, X. Cao, Z. Zhang, Impact of Long-Acting Somatostatin Analogues on Glucose Metabolism in Acromegaly: A Hospital-Based Study. Int. J. Endocrinol. (2018). 10.1155/2018/3015854 10.1155/2018/3015854 PMC5944271 29853879

[110] Colao A, Auriemma RS, Savastano S, Galdiero M, Grasso LFS, Lombardi G, Pivonello R (2009). Glucose tolerance and somatostatin analog treatment in acromegaly: A 12-month study. J. Clin. Endocrinol. Metab..

[111] Couture E, Bongard V, Maiza JC, Bennet A, Caron P (2012). Glucose status in patients with acromegaly receiving primary treatment with the somatostatin analog lanreotide. Pituitary.

[112] Cappellani D, Urbani C, Sardella C, Scattina I, Marconcini G, Lupi I, Manetti L, Marcocci C, Bogazzi F (2019). Diabetes mellitus induced by somatostatin analogue therapy is not permanent in acromegalic patients. Endocrinol. Diabetes Metab..

[113] Tanaka S, Haketa A, Yamamuro S, Suzuki T, Kobayashi H, Hatanaka Y, Ueno T, Fukuda N, Abe M, Yoshino A, Soma M (2018). Marked alteration of glycemic profile surrounding lanreotide administration in acromegaly: A case report. J. Diabetes Investig..

[114] Colao A, Bronstein MD, Freda P, Gu F, Shen CC, Gadelha M, Fleseriu M, van der Lely AJ, Farrall AJ, Hermosillo Reséndiz K, Ruffin M, Chen Y, Sheppard M (2014). Pasireotide versus octreotide in acromegaly: A head-to-head superiority study. J. Clin. Endocrinol. Metab..

[115] Gadelha MR, Bronstein MD, Brue T, Coculescu M, Fleseriu M, Guitelman M, Pronin V, Raverot G, Shimon I, Lievre KK, Fleck J, Aout M, Pedroncelli AM, Colao A (2014). Pasireotide versus continued treatment with octreotide or lanreotide in patients with inadequately controlled acromegaly (PAOLA): A randomised, phase 3 trial. Lancet Diabetes Endocrinol..

[116] M. Gadelha, M. Bex, M., Colao, A., Pedroza García, E.M., Poiana, C., Jimenez-Sanchez, M., Yener, S., Mukherjee, R., Bartalotta, A., Maamari, R., Raverot, G. Evaluation of the Efficacy and Safety of Switching to Pasireotide in Patients with Acromegaly Inadequately Controlled with First-Generation Somatostatin Analogs. Front. Endocrinol. (Lausanne). 10, (2020). 10.3389/fendo.2019.00931 10.3389/fendo.2019.00931 PMC7008501 32117045

[117] Tahara S, Murakami M, Kaneko T, Shimatsu A (2017). Efficacy and safety of long-acting pasireotide in Japanese patients with acromegaly or pituitary gigantism: results from a multicenter, open-label, randomized, phase 2 study. Endocr. J..

[118] Gadelha MR, Gu F, Bronstein MD, Brue TC, Fleseriu M, Shimon I, van der Lely AJ, Ravichandran S, Kandra A, Pedroncelli AM, Colao AAL (2020). Risk factors and management of pasireotide-associated hyperglycemia in acromegaly. Endocr. Connect.

[119] Stelmachowska-Banaś M, Czajka-Oraniec I, Tomasik A, Zgliczyński W (2022). Real-world experience with pasireotide-LAR in resistant acromegaly: a single center 1-year observation. Pituitary.

[120] M.D. Bronstein, M. Fleseriu, S. Neggers, A. Colao, M. Sheppard, F. Gu, C.C. Shen, M. Gadelha, A.J. Farrall, K.H. Reséndiz, M. Ruffin, Y.M. Chen, P. Freda, Switching patients with acromegaly from octreotide to pasireotide improves biochemical control: Crossover extension to a randomized, double-blind, Phase III study. BMC Endocr. Disord. **16**, (2016). 10.1186/s12902-016-0096-8 10.1186/s12902-016-0096-8 PMC4818908 27039081

[121] Henry RR, Ciaraldi TP, Armstrong D, Burke P, Ligueros-Saylan M, Mudaliar S (2013). Hyperglycemia associated with pasireotide: Results from a mechanistic study in healthy volunteers. J. Clin. Endocrinol. Metab..

[122] Tarasco E, Seebeck P, Pfundstein S, Daly AF, Eugster PJ, Harris AG, Grouzmann E, Lutz TA, Boyle CN (2017). Effect of AP102, a subtype 2 and 5 specific somatostatin analog, on glucose metabolism in rats. Endocrine.

[123] Higham CE, Rowles S, Russell-Jones D, Umpleby AM, Trainer PJ (2009). Pegvisomant improves insulin sensitivity and reduces overnight free fatty acid concentrations in patients with acromegaly. J. Clin. Endocrinol. Metab..

[124] Bernabeu I, Pico A, Venegas E, Aller J, Alvarez-Escolá C, García-Arnés JA, Marazuela M, Jonsson P, Mir N, García Vargas M, Spanish ACROSTUDY Group (2016). Safety of long-term treatment with Pegvisomant: analysis of Spanish patients included in global ACROSTUDY. Pituitary.

[125] Brue T, Lindberg A, Jan van der Lely A, Akerblad AC, Koltowska-Häggström M, Gomez R, Droste M, Hey-Hadavi J, Strasburger CJ, Camacho-Hübner C: (2019). Diabetes in patients with acromegaly treated with pegvisomant: observations from acrostudy. Endocrine.

[126] Guevara-Aguirre J, Rosenbloom AL, Balasubramanian P, Teran E, Guevara-Aguirre M, Guevara C, Procel P, Alfaras I, de Cabo R, di Biase S, Narvaez L, Saavedra J, Longo VD (2015). GH receptor deficiency in ecuadorian adults is associated with obesity and enhanced insulin sensitivity. J. Clin. Endocrinol. Metab..

[127] Guevara-Aguirre J, Procel P, Guevara C, Guevara-Aguirre M, Rosado V, Teran E (2016). Despite higher body fat content, Ecuadorian subjects with Laron syndrome have less insulin resistance and lower incidence of diabetes than their relatives. Growth Horm. IGF Res.

[128] Muhammad A, van der Lely AJ, Delhanty PJD, Dallenga AHG, Haitsma IK, Janssen JAMJL, Neggers SJCMM (2018). Efficacy and safety of switching to pasireotide in patients with acromegaly controlled with pegvisomant and first-generation somatostatin analogues (PAPE Study). J. Clin. Endocrinol. Metab..

[129] Lopez Vicchi F, Luque GM, Brie B, Nogueira JP, Garcia Tornadu I, Becu-Villalobos D (2016). Dopaminergic drugs in type 2 diabetes and glucose homeostasis. Pharmacol. Res..

[130] R.S. Auriemma, D. de Alcubierre, R. Pirchio, R. Pivonello, A. Colao, Glucose abnormalities associated to prolactin secreting pituitary adenomas. Front. Endocrinol. **10**, (2019). 10.3389/fendo.2019.00327 10.3389/fendo.2019.00327 PMC6540784 31191454

[131] Feek CM, Bevan JS, Taylor S, Brown NS, Baird JD (1981). The Effect of Bromocryptine on Insulin Secretion and Glucose Tolerance in Patients with Acromegaly. Clin. Endocrinol..

[132] Chiba T, Chihara K, Minamitani N, Goto B, Kadowaki S, Taminato T, Matsukura S, Fujita T (1982). Effect of Long Term Bromocriptine Treatment on Glucose Intolerance in Acromegaly. Horm. Metabol. Res..

[133] Rau H, Althoff P-H, Schmidt K, Badenhoop K, Usadel KH (1993). Bromocriptine treatment over 12 years in acromegaly: effect on glucose tolerance and insulin secretion*. Clin. Investig..

[134] Roemmler J, Steffin B, Gutt B, Schneider HJ, Sievers C, Bidlingmaier M, Schopohl J (2010). The acute effect of a single application of cabergoline on endogenous GH levels in patients with acromegaly on pegvisomant treatment. Growth Horm. IGF Res.

[135] Pijl H, Ohashi S, Matsuda M, Miyazaki Y, Mahankali A, Kumar V, Pipek R, Iozzo P, Lancaster JL, Cincotta AH, de Fronzo RA (2000). Bromocryptine. A novel approach to the treatment of type 2 diabetes. Diabetes Care.

[136] S. Störmann, J. Schopohl, Drug treatment strategies for secondary diabetes in patients with acromegaly. Exp. Opin. Pharmacother. 1883–1895 (2020). 10.1080/14656566.2020.1789098 10.1080/14656566.2020.1789098 32633582

[137] Baroni MG, Giorgino F, Pezzino V, Scaroni C, Avogaro A (2016). Italian Society for the Study of Diabetes (SID)/Italian Endocrinological Society (SIE) guidelines on the treatment of hyperglycemia in Cushing’s syndrome and acromegaly. J. Endocrinol. Invest.

[138] Cambuli VM, Galdiero M, Mastinu M, Pigliaru F, Auriemma RS, Ciresi A, Pivonello R, Amato M, Giordano C, Mariotti S, Colao A, Baroni MG (2012). Glycometabolic control in acromegalic patients with diabetes: A study of the effects of different treatments for growth hormone excess and for hyperglycemia. J. Endocrinol. Invest.

[139] Gradišer M, Matovinović M, Vrkljan M (2007). Decrease in Growth Hormone and Insulin-like Growth Factor (IGF)-1 Release and Amelioration of Acromegaly Features after Rosiglitazone Treatment of Type 2 Diabetes Mellitus in a Patient with Acromegaly Case Report Case Report. Croat. Med. J..

[140] Watanabe A, Komine F, Nirei K, Tamura K, Nabe K, Aiba N, Kamoshida S, Otsuka M, Okubo H, Kanou M, Sawada S, Uchiyama T, Nakamurat S, Arakawa Y (2004). A case of secondary diabetes mellitus with acromegaly improved by pioglitazone. Diab. Med.

[141] Zaina A, Grober Y, Abid A, Arad E, Golden E, Badarny S (2021). Sodium glucose cotransporter 2 inhibitors treatment in acromegalic patients with diabetes—a case series and literature review. Endocrine.

[142] Quarella M, Walser D, Brändle M, Fournier JY, Bilz S (2017). Rapid Onset of Diabetic Ketoacidosis after SGLT2 inhibition in a patient with unrecognized acromegaly. J. Clin. Endocrinol. Metab..

[143] Adnan Z (2019). Sodium Glucose Co-transporter Inhibitors in Patients with Acromegaly and Diabetes. Trends Endocrinol. Metab..

[144] Samson SL, Gu F, Feldt-Rasmussen U, Zhang S, Yu Y, Witek P, Kalra P, Pedroncelli AM, Pultar P, Jabbour N, Paul M, Bolanowski M (2021). Managing pasireotide-associated hyperglycemia: a randomized, open-label, Phase IV study. Pituitary.

[145] Heaney AP, Fernando M, Melmed S (2003). PPAR-γ receptor ligands: Novel therapy for pituitary adenomas. J. Clin. Invest.

[146] Kashiwagi Y, Mizuno Y, Harada E, Shono M, Morita S, Yoshimura M, Yano M, Yasue H (2013). Suppression of primary aldosteronism and resistant hypertension by the peroxisome proliferator-activated receptor gamma agonist pioglitazone. Am. J. Med Sci..

[147] Albertelli M, Nazzari E, Dotto A, Grasso LF, Sciallero S, Pirchio R, Rebora A, Boschetti M, Pivonello R, Bitti SR, Colao AAL, Ferone D (2021). Possible protective role of metformin therapy on colonic polyps in acromegaly: An exploratory cross-sectional study. Eur. J. Endocrinol..

[148] Davies MJ, Aroda VR, Collins BS, Gabbay RA, Green J, Maruthur NM, Rosas SE, del Prato S, Mathieu C, Mingrone G, Rossing P, Tankova T, Tsapas A, Buse JB (2022). Management of Hyperglycemia in Type 2 Diabetes, 2022. A Consensus Report by the American Diabetes Association (ADA) and the European Association for the Study of Diabetes (EASD). Diabetes Care.

